# The left DTI-ALPS index: a potential glymphatic mediator of cognitive and motor dysfunction in Parkinson’s disease

**DOI:** 10.3389/fmed.2025.1674718

**Published:** 2025-11-28

**Authors:** Yanyan Li, Jian Song, Yuqing Zhao, Zengru Lin, Xingduo Pan, Ming Li, Xiaoyan Zhou, Zhen Zhang, Wei Wei, Xiehua Xue

**Affiliations:** 1 Department of Medical Imaging, The Affiliated Rehabilitation Hospital, Fujian University of Traditional Chinese Medicine, Fuzhou, China; 2 Fujian Key Laboratory of Cognitive Rehabilitation, Fuzhou, China; 3 College of Rehabilitation Medicine, Fujian University of Traditional Chinese Medicine, Fuzhou, China

**Keywords:** Parkinson’s disease, Diffusion Tensor Imaging Along the Perivascular Space (DTI-ALPS), magnetic resonance imaging (MRI), cognitive function, motor function

## Abstract

**Introduction:**

This study employed the Diffusion Tensor Imaging Along the Perivascular Space (DTI-ALPS) index to evaluate glymphatic function in patients with Parkinson’s disease (PD) and investigated its association with cognitive and motor functions.

**Methods:**

Clinical data from the PD group (*n* = 64) and the healthy control group (HC, *n* = 30), matched for age, sex, and education years, were included. Based on Montreal Cognitive Assessment (MoCA) scale, PD patients were further categorized into subgroups. All participants underwent DTI scan and scale assessments.

**Results:**

Compared with the HC group, the PD group exhibited increased diffusivity of both the left projection (Dzzproj) and association fibers (Dzzassoc) along the *z*-axis (*p* < 0.05) and a reduced left DTI-ALPS index (*p* < 0.05). The left Dzzproj was negatively correlated with MoCA score (*r* = −0.299, *p* = 0.024, *q* = 0.048). The left Dzzassoc was negatively correlated with MoCA score (*r* = −0.280, *p* = 0.035, *q* = 0.035), and Movement Disorder Society-Unified Parkinson’s Disease Rating Scale III (MDS-UPDRS III) score (*r* = 0.333, *p* = 0.011, *q* = 0.022). The left DTI-ALPS index showed positive correlations with MoCA score (*r* = 0.350, *p* = 0.008, *q* = 0.015) and a negative correlation with MDS-UPDRS III score (*r* = −0.322, *p* = 0.015, *q* = 0.015). Within the PD subgroup analysis, when compared to the HC group, and throughout the progression from PD with cognition normal (PDCN) to PD with dementia (PDD), left Dzzproj (*F* = 10.240, *P* < 0.001), and left Dzzassoc (*F* = 11.060, *p* < 0.001) all demonstrated a stepwise increasing trend. Conversely, the MoCA total score (*F* = 259.985, *P* < 0.001) and the left DTI-ALPS index (*F* = 11.060, *P* < 0.001) exhibited a stepwise decreasing trend. Mediation analysis revealed that the left DTI-ALPS index mediated the effect of MoCA scores on MDS-UPDRS III scores.

**Conclusion:**

Abnormalities in the left DTI-ALPS index and diffusivity reflect underlying glymphatic system (GS) dysfunction and white matter microstructural damage in PD patients. These neuropathological changes are significantly associated with, and collectively contribute to, the progression of motor and cognitive decline. Furthermore, the left DTI-ALPS index shows promise as a novel biomarker for identifying cognitive impairment in PD patients.

## 1 Introduction

Parkinson’s disease (PD) is the second most common neurodegenerative disorder. Its core pathological features include the degeneration of dopaminergic neurons in the midbrain substantia nigra, and the formation of intracellular Lewy bodies within surviving neurons. These pathological hallmarks are primarily attributed to the dysregulation of α-synuclein production and clearance, resulting in its abnormal aggregation (1). Consequently, PD patients manifest severe motor deficits, such as tremor and bradykinesia. These impairments not only significantly diminish patients’ quality of life but also impose a substantial burden on society (2).

Notably, cognitive impairment, a crucial non-motor symptom of PD, significantly accelerates disability progression (3). The co-existence of motor and cognitive impairments suggests shared underlying pathological mechanisms. Their synergistic effect further exacerbates patients’ overall functional decline. Therefore, elucidating the intrinsic link and shared pathological basis between cognitive and motor impairments in PD, and identifying quantifiable, non-invasive neuroimaging biomarkers, are crucial for early diagnosis and intervention in PD.

With the deepening understanding of PD pathogenesis, a crucial cerebral waste clearance system-the glymphatic system (GS)-has garnered increasing attention (4, 5). Studies have shown that GS dysfunction leads to the abnormal accumulation of toxic proteins in the brain, further exacerbating neuronal degeneration (6) and playing a crucial role in both motor and cognitive impairments. This finding offers a new perspective for understanding the complex clinical manifestations of PD: both motor deficits and cognitive decline may be closely linked to impaired GS clearance function and associated protein metabolic disturbances. However, *in vivo* non-invasive assessment of GS functional status in PD patients remains challenging. Therefore, developing a reliable, non-invasive method for assessing GS function is of paramount importance.

Diffusion Tensor Imaging along the Perivascular Space (DTI-ALPS) is a novel non-invasive technique for assessing glymphatic function. This non-contrast-enhanced technique utilizes diffusion imaging sequences to measure the diffusivity of water molecules along the *x*, *y*, and *z* axes within the projection and association fibers of the periventricular white matter. The resulting data are then used to calculate the DTI-ALPS index, which can, to some extent, reflect the functional status of the glymphatic system (7). Furthermore, studies have demonstrated a significant correlation between the DTI-ALPS index and glymphatic function measured via intrathecal injection, indicating its ability to reflect glymphatic clearance capacity with high accuracy and reliability (8). Moreover, studies have revealed that the DTI-ALPS index is significantly lower in PD patients compared to healthy controls (HC), and that this index is also significantly correlated with the severity of motor symptoms and the overall cognitive status (9).

However, the interpretation of the DTI-ALPS index is facing new challenges. A recent post-mortem study revealed that a reduced DTI-ALPS index not only reflects glymphatic dysfunction but also indicates asymmetry in white matter microstructure due to α-synuclein deposition (10). This finding suggests that the DTI-ALPS index reflects both glymphatic function and microstructural integrity. In this context, two critical gaps remain in the current research. First, in the field of cognitive research in PD, existing studies have predominantly focused on either compared PD patients with mild cognitive impairment (PD-MCI) to healthy controls (11) or used the DTI-ALPS index to predict the conversion from PD-MCI to PD with dementia (PDD) (3). A systematic investigation across different cognitive strata [e.g., PD with cognition normal (PDCN), PD-MCI, and PDD] is lacking. It remains unclear at which stage of cognitive decline glymphatic dysfunction, microstructural changes, and asymmetry become prominent, and whether the extent of this impairment strictly corresponds to the cognitive stratification. Second, the vast majority of studies have focused solely on the final DTI-ALPS index, while overlooking the diffusivities along the *x*, *y*, and *z* axes (Dxx, Dyy, Dzz) in projection and association fibers. This has prevented a deeper understanding of the underlying microphysiological and pathological mechanisms driving the DTI-ALPS index in patients with PD.

Therefore, this study aims to utilize the DTI-ALPS index to evaluate GS function in PD patients and to investigate its relationship with cognitive and motor functions. First, we not only analyze the DTI-ALPS index but also examined the directional diffusivities of projection and association fibers, exploring their correlations with motor and cognitive performance. Second, we observe the specific patterns of change in the DTI-ALPS index and directional diffusivities across different cognitive subgroups (PDCN, PD-MCI, and PDD). Finally, this study analyze the interrelationships among the DTI-ALPS index, motor function, and cognitive function to provide a scientific basis for the future development of DTI-ALPS-based diagnostic and therapeutic strategies for PD.

## 2 Materials and methods

### 2.1 Participants

This study was conducted at the Rehabilitation Hospital Affiliated to Fujian University of Traditional Chinese Medicine from April to June 2025. A total of 64 patients with PD (37 males and 27 females) were enrolled. Additionally, 30 healthy controls (12 males and 18 females), who were matched to the PD group in terms of age, gender, and education level, were recruited. This study protocol was approved by the Ethics Committee of the Rehabilitation Hospital affiliated with the Fujian University of Traditional Chinese Medicine (No:2025YJS-027-02), and written informed consent was obtained from all participants.

Inclusion criteria for the PD group: (1) A diagnosis of PD based on the Movement Disorder Society (MDS) criteria (12). (2) Participants aged between 45 and 80 years, right-handedness. (3) All assessments and MRI scans were conducted while patients were in the “ON” medication state. (4) Ability to cooperate with and complete all clinical and imaging assessments.

Exclusion criteria for the PD group: (1) Presence of other neurological or psychiatric diseases that could affect cognitive or motor function. (2) Cerebral small vessel disease (e.g., lacunar infarction, multiple cerebral microbleeds) or stroke. (3) Contraindications for magnetic resonance imaging (MRI).

Inclusion criteria for the healthy control group: (1) No evidence of cerebral small vessel disease as confirmed by MRI and no history of neurological or psychiatric disorders. (2) Cognitively normal, as determined by a Montreal Cognitive Assessment (MoCA) score of ≥26 after adjustment for education level. (3) Aged between 45 and 80 years, right-handedness. (4) No contraindications for MRI.

### 2.2 Clinical scale assessment

#### 2.2.1 Montreal cognitive assessment (MoCA)

The MoCA is a 30-point cognitive screening tool where higher scores indicate better overall cognitive function (13). Based on MoCA scores, PD patients were categorized into three cognitive subgroups: the PDCN group (MoCA score ≥26), the PD-MCI group (MoCA score 19–25), and the PDD group (MoCA score ≤18) (14).

#### 2.2.2 Movement Disorder Society-Unified Parkinson’s Disease Rating Scale (MDS-UPDRS)

The MDS-UPDRS is a comprehensive tool used to assess the severity and progression of PD across non-motor and motor symptoms (15). Part I assesses non-motor experiences of daily living (score range 0-52 points), where higher scores indicate more severe non-motor symptoms and associated daily functional impairment. Part II evaluates motor experiences of daily living (score range 0-52 points). Part III provides an objective motor examination to assess the severity of motor dysfunction (score range 0-132 points). For both Part II and Part III, higher scores signify more severe motor dysfunction.

### 2.3 MRI scan protocol and analysis

#### 2.3.1 MRI data acquisition

MRI data were acquired at the Department of Imaging, Rehabilitation Hospital Affiliated to Fujian University of Traditional Chinese Medicine. All participants underwent MRI scanning using a 3.0T Prisma MRI scanner (Siemens, Germany) equipped with a 64-channel head coil.

The imaging protocol included T1-weighted imaging (T1WI), T2 fluid-attenuated inversion recovery (T2 FLAIR), and diffusion tensor imaging (DTI). The DTI sequence employed a single-shot echo planar imaging (SS-EPI) technique with 30 acquisition directions and two *b*-values: *b* = 0 and *b* = 1,000 s/mm^2^. Detailed parameters are provided in Supplementary materials Table 1.

#### 2.3.2 DTI-ALPS processing and measurement

Raw DTI images were preprocessed using the FMRIB Software Library (FSL, version 5.0.9) in a Linux OS environment (Oracle VM VirtualBox). Preprocessing steps included format conversion, head motion and eddy current correction, gradient direction correction, and skull stripping. The Dtifit command was then used to generate directional diffusivity maps (Dxx, Dyy, and Dzz) along the *x*, *y*, and *z* axes, respectively. Regions of interest (ROIs) were manually drawn for each subject using ITK-SNAP software. These ROIs were placed in the bilateral periventricular regions, specifically targeting projection and association fiber tracts to assess diffusivity along the *x*, *y*, and *z* axes. Following this, the mean DTI-ALPS indices for the left and right hemispheres, as well as the whole brain, were calculated.

**Figure 1:**
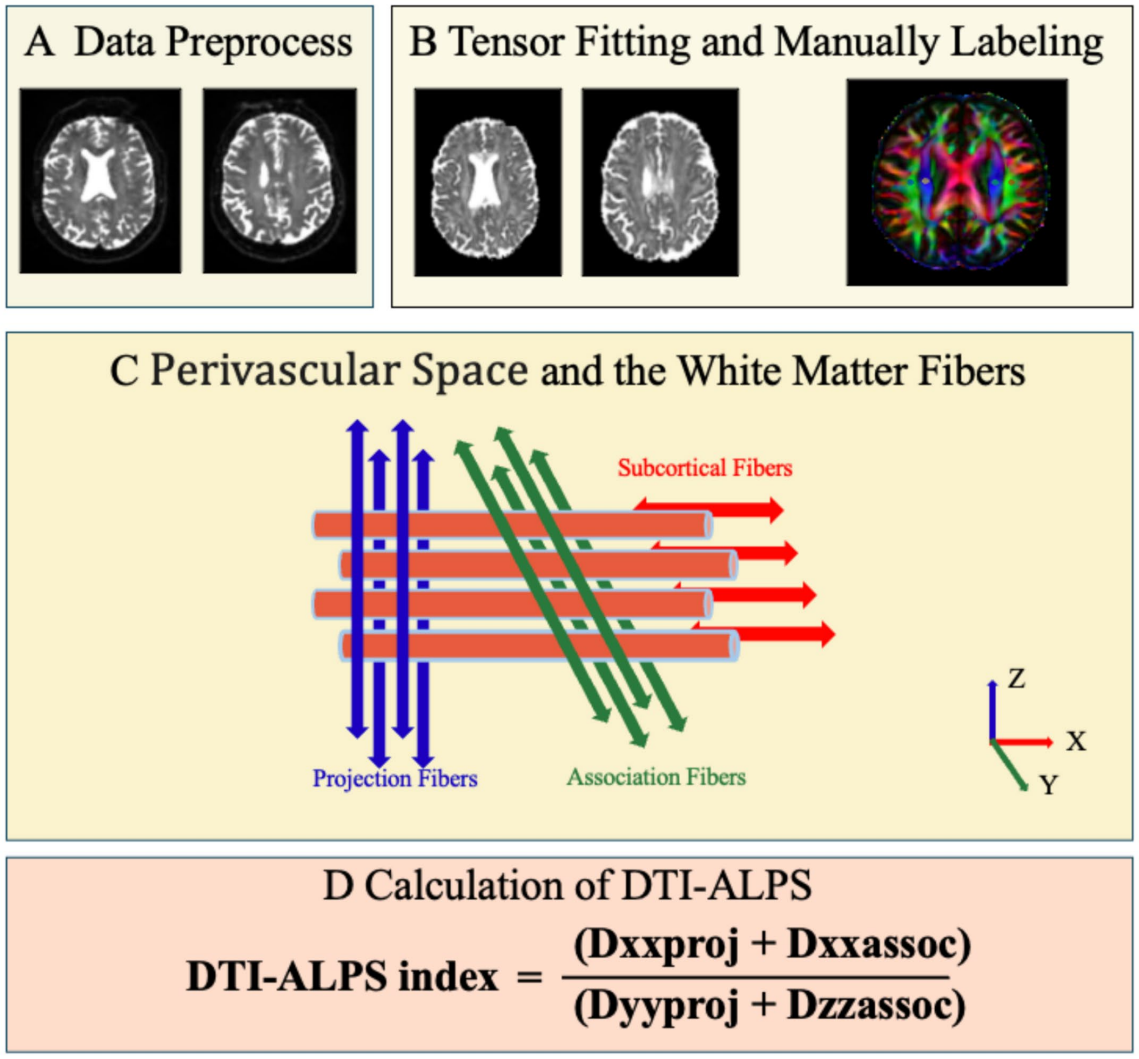
DTI-ALPS data analysis flowchart. **(A)** DTI data preprocess. **(B)** Tensor fitting and manually labeling. **(C)** Perivascular space and the white matter fibers. **(D)** Calculation of DTI-ALPS.

At the lateral ventricular level, fluid movement within the perivascular spaces (PVS) is orthogonal to the ventricular wall, aligning predominantly with the *x*-axis. This flow is also orthogonal to both the projection fibers (primarily *z*-axis) and association fibers (primarily *y*-axis), forming a mutually orthogonal system. Consequently, in this study, ROIs for projection and association fibers were delineated on the superior corona radiata (SCR) and superior longitudinal fasciculus (SLF), respectively. The ALPS-index was calculated following established protocols by determining the diffusivity along the *x*, *y*, and *z* axes within each ROI (16), see Figure 1.

### 2.4 Statistical analysis

Statistical analysis were performed using SPSS software (version 26.0). Count data are presented as frequencies (percentages), and the chi-square test was used to compare differences between groups. For measurement data, normality was first assessed. Normally distributed data were expressed as mean ± standard deviation, while non-normally distributed data were presented as median (interquartile range, IQR). A *p*-value of <0.05 was considered to indicate statistical significance.

For comparisons between the PD group and the HC group, normally distributed data were analyzed using an independent samples *t*-test. Non-normally distributed data were compared using a non-parametric rank sum test (Mann–Whitney U test).

For comparisons among the PD cognitive subgroups and the HC group, normally distributed data were analyzed using one-way ANOVA for the four groups (PDCN, PDMCI, PDD, and HC groups). For non-normally distributed data, a non-parametric rank sum test (Kruskal–Wallis test) was used for comparison. Post-hoc multiple comparisons were performed using Bonferroni correction.

Partial correlation analysis was performed to examine the correlation between the DTI-ALPS index and MoCA scores, as well as MDS-UPDRS I to III scale scores, while controlling for age, gender, education years, Hoehn and Yahr (H-Y) stage, duration of disease, PD side-of-onset, and Levodopa equivalent dose.

The results of the partial correlation analysis were corrected for multiple comparisons using the False Discovery Rate (FDR) method. In this study, both the uncorrected *p*-values and the FDR-corrected *q*-values are reported, with a *q*-value of less than 0.05 considered to indicate statistical significance.

Variables that were significant in the partial correlation analysis were selected for mediated moderation model analysis. After controlling for participants’ age, gender, and educational years, this study used the PROCESS macro (V4.1) in SPSS to estimate the mediating effects of the DTI-ALPS index on cognitive function. Analyses of the mediated moderation model were based on ordinary least squares regression with nonparametric bootstrap procedures (5,000 trials), yielding bias-corrected confidence intervals (CIs) for effect size inference. *p* < 0.05 was considered statistically significant (a significant effect was indicated if the 95% CI did not cover the null).

## 3 Results

### 3.1 Clinical demographic characteristics of PD group and HC group

The PD group and the HC group showed no statistically significant differences in age, gender, or education years (*p* > 0.05). Conversely, a significant difference was found in the total MoCA score (*p* < 0.001), see Table 1.

**Table 1:** Demographic and clinical data and scale scores for PD group and control group.

Item	PD group (*n* = 64)	PDCN group (*n* = 26)	PDMCI group (*n* = 25)	PDD group (*n* = 13)	HC group (*n* = 30)	t/Z/*x^2^*	*p*
Age (years)	70.00 (64.00, 74.00)	68.50 (61.75, 73.00)	72.00 (64.50, 75.00)	70.00 (66.50, 77.00)	67.50 (65.00, 72.25)	−0.744	0.460
Gender (male/female)	37/27	16/10	15/10	6/7	12/18	2.597	0.125
Education years (years)	12.00 (9.00, 12.00)	12.00 (7.5, 12.00)	12.00 (9.00, 12.00)	12.00 (9.00, 12.00)	9.00 (9.00, 12.00)	−0.952	0.344
HY stage	3.00 (1.00, 3.00)	2.00 (1.00, 2.25)	3.00 (1.50, 3.00)	3.00 (3.00, 3.00)	—	—	—
Duration of disease (years)	4.75 (3.00, 7.00)	3.00 (2.00, 5.25)	4.50 (3.50, 5.75)	8.00 (6.50, 8.00)	—	—	—
PD side-of-onset (left/right)	20/44	8/18	8/17	4/9	—	—	—
Levodopa equivalent dose (mg/day)	375.00 (187.50, 375.00)	187.50 (187.50, 375.00)	375.00 (187.50, 375.00)	750.00 (625.00, 1000.00)	—	—	—
MoCA score	24.50 (21.00, 28.00)	28.00 (26.75, 29.00)	22.00 (21.00, 24.50)	14.00 (12.00, 17.50)	28.00 (28.00, 29.00)	−5.089	**<0.001**
MDS-UPDRS I score	7.00 (4.00, 17.50)	6.00 (3.75, 9.00)	8.00 (4.00, 13.50)	8.00 (6.50, 13.50)	—	—	—
MDS-UPDRS II score	11.66 ± 6.07	10.12 ± 4.28	11.56 ± 6.41	14.92 ± 7.49	—	—	—
MDS-UPDRS III score	33.19 ± 13.21	29.92 ± 10.73	33.20 ± 14.50	39.69 ± 13.67	—	—	—

PD, Parkinson’s disease; PDCN, Parkinson’s disease with cognition normal; PDMCI, Parkinson’s disease with mild cognitive impairment; PDD, Parkinson’s disease dementia; HC, healthy control; MoCA, Montreal Cognitive Assessment; MDS-UPDRS, Movement Disender Soaiety-Unified Parkinson Disease Rating Scale. Values in bold denote statistically significant results (*p* < 0.05).

### 3.2 The inter-group comparison of the DTI-ALPS index between the PD group and the HC group

Compared with the HC group, the PD group exhibited higher diffusivity in the left association fibers along the *x*-axis (Dxxassoc, *p* = 0.033); in the left projection fibers along the *z*-axis (Dzzproj, *p* = 0.002); in the left association fibers along the *z*-axis (Dzzassoc, *p* < 0.001); in the right association fibers along the *x*-axis (Dxxassoc, *p* = 0.042); and in the right projection fibers along the *z*-axis (Dzzproj, *p* = 0.013). Additionally, the left DTI-ALPS index was lower in the PD group (*p* = 0.002). The other indicators were not statistically significant, see Table 2.

**Table 2:** The differences of DTI-ALPS index and diffusion rate between PD group and control group.

Item	PD (*n* = 64)	PDCN (*n* = 26)	PDMCI (*n* = 25)	PDD (*n* = 13)	HC (*n* = 30)	t/Z	*p*
The diffusion rate of the left projection fibers along the *x*-axis (Left Dxxproj) (10^−3^ mm^2^/s)	(0.60 ± 0.07)	0.60 (0.60,0.70)	0.60 (0.60,0.60)	0.60 (0.60,0.70)	(0.60 ± 0.05)	−0.124	0.902
The diffusion rate of the left association fibers along the *x*-axis (Left Dxxassoc) (10^−3^ mm^2^/s)	(0.70 ± 0.09)	(0.70 ± 0.08)	(0.70 ± 0.10)	(0.70 ± 0.08)	(0.70 ± 0.06)	2.172	**0.033**
The diffusion rate of the left projection fibers along the *y*-axis (Left Dyyproj) (10^−3^ mm^2^/s)	0.50 (0.40, 0.50)	0.50 (0.50, 0.50)	0.50 (0.40, 0.50)	0.50 (0.50, 0.60)	0.50 (0.40, 0.50)	−1.821	0.069
The diffusion rate of the left association fibers along the *y*-axis (Left Dyyassoc) (10^−3^ mm^2^/s)	(1.10 ± 0.08)	(1.10 ± 0.07)	(1.10 ± 0.08)	(1.20 ± 0.08)	(1.10 ± 0.08)	0.327	0.745
The diffusion rate of the left projection fibers along the *z*-axis (Left Dzzproj) (10^−3^ mm^2^/s)	(1.20 ± 0.13)	(1.10 ± 0.10)	(1.20 ± 0.10)	(1.20 ± 0.16)	(1.10 ± 0.07)	3.248	**0.002**
The diffusion rate of the left association fibers along the *z*-axis (Left Dzzassoc) (10^−3^ mm^2^/s)	0.40 (0.40, 0.50)	(0.04 ± 0.05)	(0.04 ± 0.07)	(0.05 ± 0.09)	0.40 (0.30, 0.40)	−4.165	**<0.001**
The diffusion rate of the right projection fibers along the *x*-axis (Right Dxxproj) (10^−3^ mm^2^/s)	0.60 (0.50, 0.70)	0.60 (0.60, 0.70)	0.60 (0.50, 0.60)	0.60 (0.06, 0.70)	0.60 (0.60, 0.60)	−0.625	0.536
The diffusion rate of the right association fibers along the *x*-axis (Right Dxxassoc) (10^−3^ mm^2^/s)	(0.70 ± 0.09)	(0.70 ± 0.09)	(0.70 ± 0.09)	(0.70 ± 0.10)	(0.70 ± 0.06)	2.065	**0.042**
The diffusion rate of the right projection fibers along the *y*-axis (Right Dyyproj) (10^−3^ mm^2^/s)	0.50 (0.40,0.50)	0.50 (0.40,0.50)	0.40 (0.40,0.50)	0.50 (0.40,0.50)	0.50 (0.40,0.50)	−0.320	0.752
The diffusion rate of the right association fibers along the *y*-axis (Right Dyyassoc) (10^−3^ mm^2^/s)	(1.10 ± 0.08)	(1.10 ± 0.06)	(1.10 ± 0.09)	(1.10 ± 0.07)	(1.10 ± 0.07)	0.011	0.991
The diffusion rate of the right projection fibers along the *z*-axis (Right Dzzproj) (10^−3^ mm^2^/s)	1.20 (1.10, 1.20)	1.10 (1.00, 1.20)	1.20 (1.10, 1.30)	1.20 (1.10, 1.20)	1.10 (1.10, 1.20)	−2.458	**0.013**
The diffusion rate of the right association fibers along the *z*-axis (Right Dzzassoc) (10^−3^ mm^2^/s)	0.40 (0.40, 0.50)	0.40 (0.40, 0.40)	0.40 (0.40, 0.50)	0.50 (0.40, 0.50)	0.40 (0.40, 0.40)	−0.937	0.352
Left DTI-ALPS index	1.42 (1.29, 1.52)	1.48 (1.35, 1.57)	1.41 (1.24, 1.53)	1.35 (1.21, 1.40)	1.51 (1.40, 1.63)	−3.034	**0.002**
Right DTI-ALPS index	1.45 (1.28, 1.58)	1.48 (1.38, 1.60)	1.37 (1.26, 1.58)	1.39 (1.20, 1.55)	1.40 (1.32, 1.57)	−0.195	0.850
Whole DTI-ALPS index	1.43 (1.34, 1.53)	1.47 (1.38, 1.57)	1.41 (1.25, 1.52)	1.36 (1.21, 1.48)	1.45 (1.37, 1.60)	−1.338	0.183

PD, Parkinson’s disease; PDCN, Parkinson’s disease with normal cognition; PDMCI, Parkinson’s disease with mild cognitive impairment; PDD, Parkinson’s disease dementia; HC, healthy control; DTI-ALPS, Diffusion Tensor Imaging Along the Perivascular Space. Values in bold denote statistically significant results (*p* < 0.05).

### 3.3 Correlation analysis of the DTI-ALPS index with cognitive function and motor function in the PD group

Partial correlation analysis, controlling for age, gender, education years, Hoehn and Yahr (H-Y) stage, duration of disease, PD side-of-onset, and Levodopa equivalent dose was performed in the PD group to examine the relationships between diffusion metrics and clinical scores.

The left Dzzproj was negatively correlated with both MoCA score (*r* = −0.299, *p* = 0.024, *q* = 0.048). The left Dzzproj was not significantly correlated with MDS-UPDRS III scores (*r* = 0.248, *p* = 0.062, *q* = 0.062).

The left Dzzassoc was negatively correlated with MoCA score (*r* = −0.280, *p* = 0.035, *q* = 0.035). Furthermore, the left Dzzassoc was positively correlated with MDS-UPDRS III score (*r* = 0.333, *p* = 0.011, *q* = 0.022).

**Figure 2:**
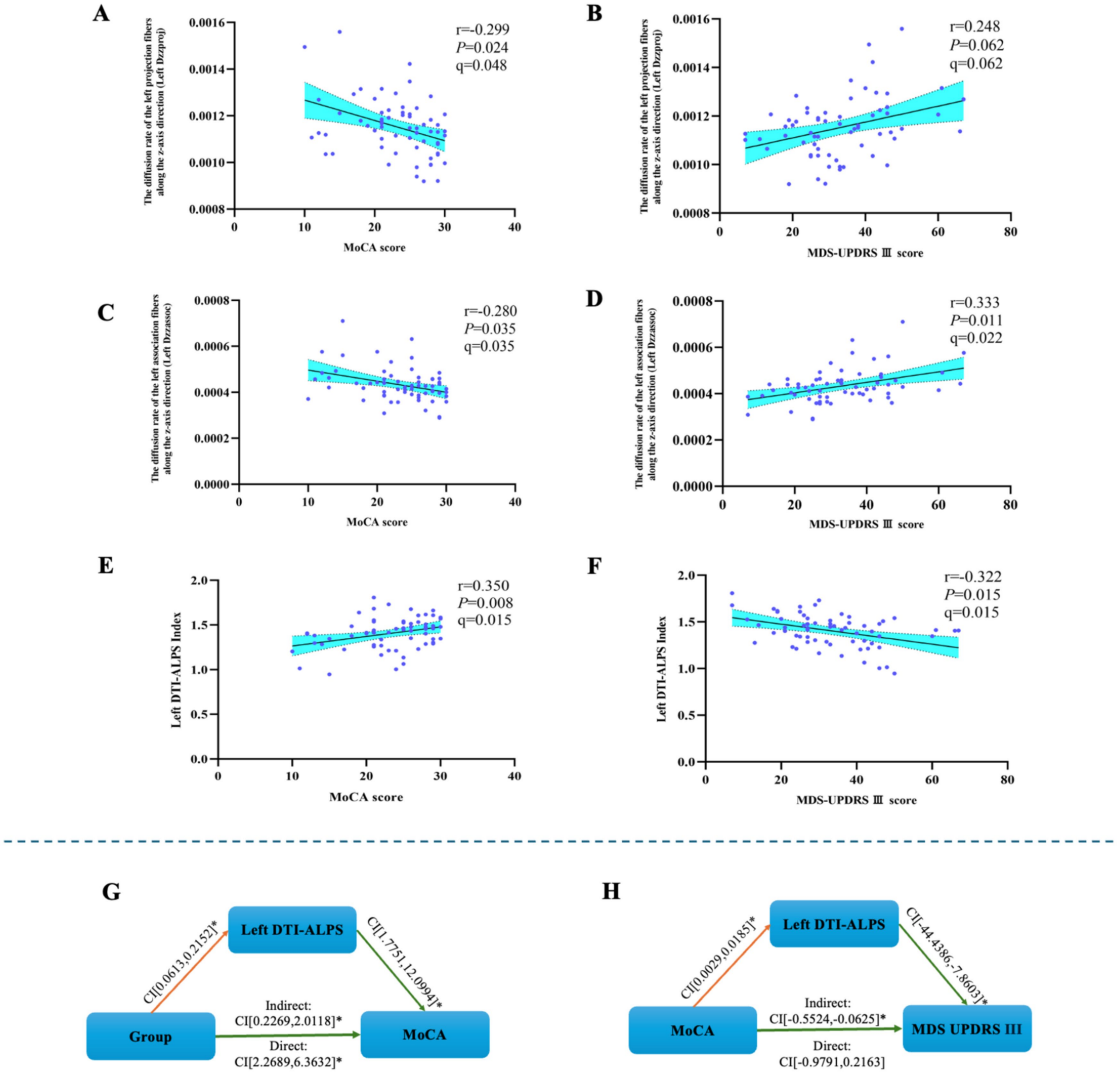
The results of the mediated moderation model analysis and the correlation analysis. **(A)** The left Dzzproj was negatively correlated with MoCA score. **(B)** The left Dzzproj was not significantly correlated with MDS-UPDRS III scores. **(C)** The left Dzzassoc was negatively correlated with MoCA score. **(D)** The left Dzzassoc was positively correlated with MDS-UPDRS III score. **(E)** The left DTI-ALPS index was positively correlated with MoCA score. **(F)** The left DTI-ALPS index was negatively correlated with MDS-UPDRS III score. **(G)** The mediated moderation model constructed with group as the independent variable (64 PD patients and 30 healthy controls), MoCA score as the dependent variable, and left DTI-ALPS as a mediator variable. **(H)** The mediated moderation model constructed with MoCA score as the independent variable (64 PD patients), MDS-UPDRS III score as the dependent variable, and left DTI-ALPS as a mediator variable.

The left DTI-ALPS index exhibited a positive correlation with MoCA score (*r* = 0.350, *p* = 0.008, *q* = 0.015). Conversely, a negative correlation was observed between the left DTI-ALPS index and MDS-UPDRS III score (*r* = −0.322, *p* = 0.015, *q* = 0.015), see Figures 2A–F.

### 3.4 The results of the mediation moderation model analysis

The mediation-moderation analysis was conducted with group as the independent variable and MoCA score as the dependent variable to examine the relationship between the left DTI-ALPS index and cognitive performance.

The MoCA score was used as the independent variable, with MDS-UPDRS III score as the dependent variable, to assess the relationship between the left DTI-ALPS index and motor performance.

The left DTI-ALPS index served as a mediator for the effect of group on MoCA score [indirect effect 95% CI: (0.2269–2.0118)] and for the effect of MoCA score on MDS-UPDRS III score [indirect effect 95% CI: (−0.5524, −0.0625)], respectively, see Figures 2G,H.

### 3.5 The inter-group comparison of the MoCA score between the PD subgroup and the HC group

In the subgroup analysis, the result revealed significant overall differences in MoCA score (*F* = 259.985, *p* < 0.001).

**Figure 3:**
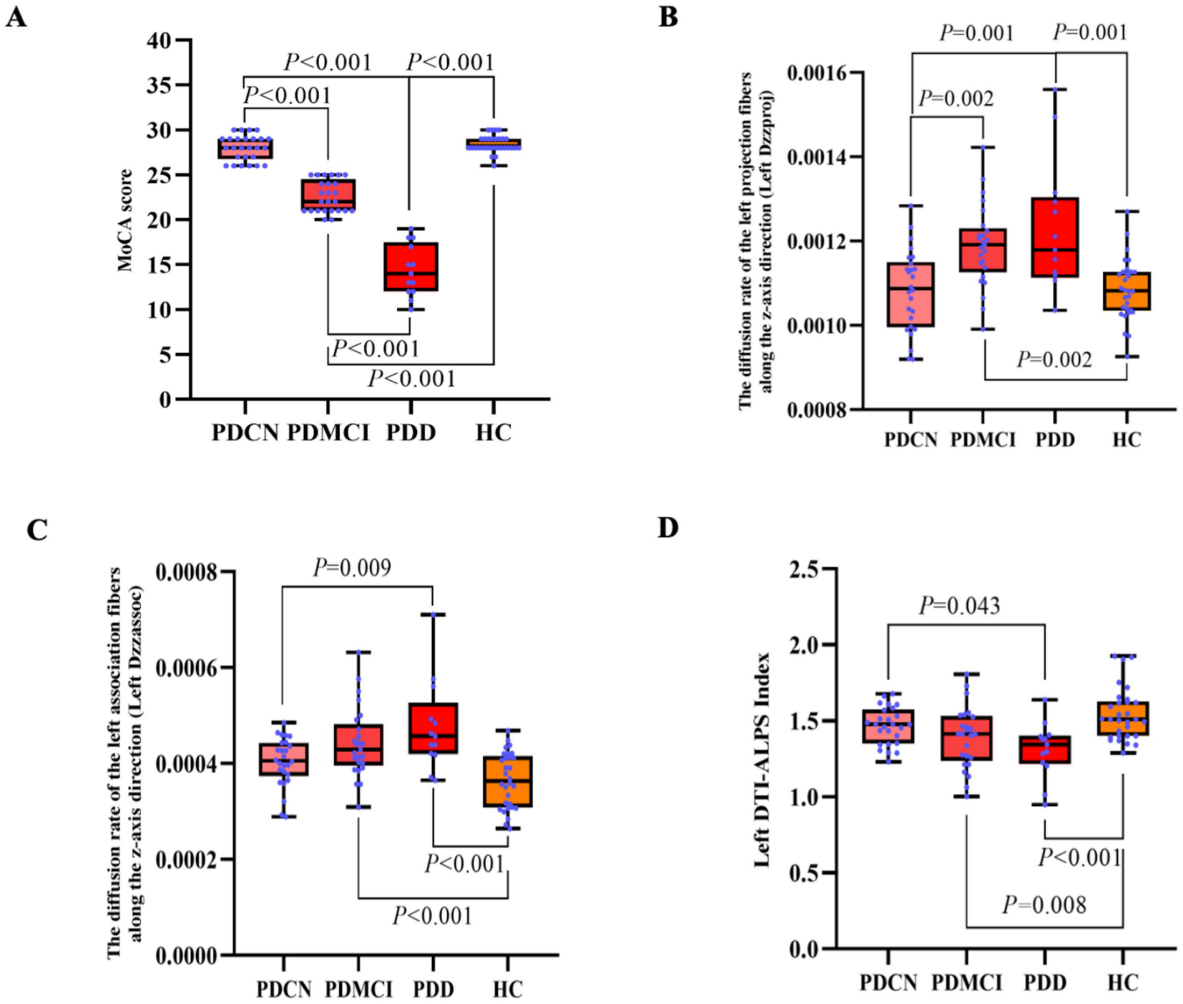
The results of the DTI-ALPS index and the MoCA score in PD subgroup and HC group. **(A)** Inter-group comparison of MoCA score among PDCN, PDMCI, PDD, and HC groups. **(B)** Inter-group comparison of left Dzzproj among PDCN, PDMCI, PDD, and HC groups. **(C)** Inter-group comparison of left Dzzassoc among PDCN, PDMCI, PDD, and HC groups. **(D)** Inter-group comparison of the left DTI-ALPS index among PDCN, PDMCI, PDD, and HC groups.

Generally, both the PDMCI and PDD groups exhibited significantly lower scores across MoCA score when compared to the PDCN group (*p* < 0.05). Similarly, compared to HC group, both PDMCI and PDD groups showed significantly decreased in MoCA score (*p* < 0.05). A pattern of progressive cognitive decline was observed, with the PDD group performing significantly decreased than the PDMCI group in MoCA score (*p* < 0.05), see Figure 3A.

### 3.6 The inter-group comparison of the DTI-ALPS index between the PD subgroup and the HC group

In the subgroup analysis, the result revealed significant differences among the groups in the left Dzzproj (*F* = 10.240, *p* < 0.001), the left Dzzassoc (*F* = 11.060, *p* < 0.001), and the left DTI-ALPS index (*F* = 11.060, *p* < 0.001).

In pairwise comparisons, the left Dzzassoc was higher in the PDD group compared to the PDCN group (*p* = 0.009). Compared to the HC group, both the PDMCI and PDD groups exhibited higher diffusivity in the left Dzzassoc (both *p* < 0.001).

For the left Dzzproj, both the PDMCI (*p* = 0.002) and PDD (*p* = 0.001) groups showed higher diffusivity compared to the PDCN group. Additionally, compared to the HC group, both the PDMCI group (*p* = 0.002) and PDD group (*p* = 0.001) also exhibited higher diffusivity in the left Dzzproj.

The PDD group exhibited a lower the left DTI-ALPS index compared to the PDCN group (*p* = 0.043). Compared to the HC group, both the PDMCI group (*p* = 0.008) and the PDD group (*p* < 0.001) showed a lower DTI-ALPS index, see Figures 3B–D.

## 4 Discussion

This study found that, compared to the HC group, PD patients exhibited increased left Dzzproj and left Dzzassoc, and these changes correlated with cognitive and motor functions. The left DTI-ALPS index in PD patients exhibited a significant decrease, which was also associated with cognitive and motor functions. Secondly, within the PD subgroups, the left Dzzproj and left Dzzassoc exhibited a progressive increase with worsening cognitive impairment (from PDCN to PDD). Conversely, the left DTI-ALPS index exhibited a progressive decrease with worsening cognitive impairment (from PDCN to PDD). Finally, the mediation model analysis showed that the effect of cognitive function on motor function was significantly mediated by the left DTI-ALPS index. These results suggest that changes in the DTI-ALPS index and other diffusion indicators in PD patients may be related to dysfunction of the glymphatic system dysfunction and may constitute the pathological basis for the progression of motor symptoms and cognitive decline.

In the PD group, both the left Dzzproj and left Dzzassoc were increased. According to the DTI-ALPS index formula, the diffusivity along the z-axis of the association fibers (Dzzassoc) serves as the denominator; therefore, an increase in this value leads to a decrease in the left DTI-ALPS index. This increased diffusivity in the projection and association fibers correlated with both motor and cognitive functions, suggesting underlying damage to the white matter microstructure. The correlation of these diffusion metrics with clinical functions may, in turn, indirectly reflect impaired GS function. This interpretation is supported by recent research indicating that the DTI-ALPS index reflects not only the flow dynamics of the glymphatic system but also the microstructural integrity of white matter fibers (10).

In this study, the ROIs for projection fibers and association fibers were placed in the middle SCR at the level of the lateral ventricle and the SLF, respectively. On one hand, the SCR is a radial white matter fiber structure extending from the internal capsule to the cerebral cortex; damage to the fiber bundles in this region may affect attention and executive function (17). Research suggests that the left SCR is an independent risk factor for PD, and degenerative damage may exist in early PD (18). On the other hand, the SLF primarily connects cortical regions such as the prefrontal lobe, parietal lobe, and occipital lobe, participating in higher cognitive functions and motor control (19). Studies indicate that microstructural abnormalities of the SLF are closely related to cognitive decline and motor symptoms in PD patients (20). Therefore, we speculate that the increase in the left Dzzproj and left Dzzassoc in PD patients, to some extent, reflects white matter damage in these fibers (21). Previous research has found that during the cognitive progression across PDCN, PD-MCI, and PDD, the volume of white matter hyperintensities (WMH) gradually increases and is closely correlated with cognitive function (22). Consistent with this, our results reveal a stepwise increase in the diffusivity of the left Dzz projection and association fibers across the PDCN, PDMCI, and PDD groups, suggesting a progression of white matter damage in patients with PD.

This study found that the left DTI-ALPS index differed significantly in PD patients and PD subgroups. Previous studies also found an abnormal left DTI-ALPS index in PD patients (23), consistent with the results of this study. Studies have reported that the DTI-ALPS index in PD patients is lower than in the HC group (24–26), and longitudinal studies have also confirmed a significant association between the DTI-ALPS index and PD clinical progression (11). The left-sided nature of our findings may relate to the characteristic asymmetries of PD, such as those in motor symptoms and network connectivity (27, 28). Since all PD patients in this study were right-handed, their left hemisphere is the dominant hemisphere. The left hemisphere dominates higher cognitive functions such as language and is more sensitive to damage (29), making it more vulnerable to degenerative changes (30). This heightened vulnerability may explain why a reduction in GS clearance efficiency, and consequently the left DTI-ALPS index, is more pronounced in the left hemisphere.

Furthermore, the majority of patients’ symptoms originated on the right side, which supports the speculation that the initial deposition of α-synuclein occurred predominantly in the left hemisphere. Research has found that the left hemisphere is more susceptible to pathological propagation and accumulation (31). A high pathological burden of alpha-synuclein could reduce the GS clearance efficiency in the left cerebral hemisphere, wherein a decrease in the left DTI-ALPS index would indicate glymphatic dysfunction. A prior study found that individuals who converted from PD-MCI to PDD had a lower left DTI-ALPS index (3). This finding corroborates the results of our study, which showed a stepwise decrease in the left DTI-ALPS index across the PDCN, PD-MCI, and PDD groups.

In the PD group, the left DTI-ALPS index was positively correlated with MoCA, and negatively correlated with MDS-UPPDRS III scores. Previous research has shown that the DTI-ALPS index is related to cognitive level (2), which is consistent with the findings of this study. The left hemisphere is dominant for higher cognitive functions and is more vulnerable to injury. This may explain the association observed between the decrease in the left DTI-ALPS index and overall cognitive function. Chen et al. used the DTI-ALPS index to evaluate GS function in PD patients and found that a decrease in the index was significantly negatively correlated with elevated plasma nuclear DNA levels (an oxidative stress marker) and significantly positively correlated with cognitive function (MMSE score) (22). This result suggests that GS dysfunction in PD patients may exacerbate oxidative stress, promoting neuronal damage or death, thereby leading to a decline in cognitive and motor functions.

Furthermore, brain white matter is extremely sensitive to hypoxia, inflammation, and metabolic damage, and its integrity relies on the GS to maintain interstitial fluid flow to promote metabolic waste exchange (32). In addition, the study by Qin et al. (21) also confirmed the negative correlation between the DTI-ALPS index and MDS-UPDRS III. The results of this study highlight its clinical value as a biomarker for cognitive and motor functions in PD.

In the PD subgroup analysis, we observed several key findings. Firstly, compared to the HC group, during the progression from PDCN to PDD, the left Dzzproj and left Dzzassoc exhibited a stepwise increasing trend, while the left DTI-ALPS index showed a stepwise decreasing trend. Secondly, both the PDMCI and PDD groups demonstrated statistically significant differences in the left Dzzproj, left Dzzassoc, and the left DTI-ALPS index when compared to the HC group. Finally, the PDD group also exhibited statistically significant differences in the left Dzzproj, left Dzzassoc, and the left DTI-ALPS index when compared to the PDCN group. Previous studies have identified the left DTI-ALPS index as a potential non-invasive biomarker for the risk of dementia conversion in PD patients (2). Collectively, these results suggest that the left DTI-ALPS index and diffusivity may serve as valuable potential biomarkers for identifying cognitive decline in PD.

Results from the mediation moderation model analysis indicate that the left DTI-ALPS index is associated with both cognitive and motor functions and mediates their interaction. This suggests that the left DTI-ALPS index, reflecting left hemispheric GS function, serves as a critical hub for cognitive-motor co-impairment. GS dysfunction may contribute to cognitive and motor impairment by exacerbating oxidative stress responses (24), promoting neuronal damage or death, and causing widespread white matter microstructural damage (33).

GS dysfunction results in extensive white matter microstructural damage, characterized by axonal loss, demyelination, and reactive astrogliosis (34). PD patients exhibit white matter microstructural damage in widespread regions such as the corpus callosum and SLF (18), along with impaired motor conduction pathways like the corticostriatal tract (33), contributing to cognitive and motor dysfunction in these patients. This study findings, including increased left Dzzproj and left Dzzassoc and a decreased left DTI-ALPS index within PD subgroups, further corroborate white matter damage within cognitive and motor pathways. Consequently, glymphatic dysfunction and microstructural damage to projection or association white matter fibers in PD patients, leading to the accumulation of toxic proteins, synergistically drive the progression of both motor and cognitive impairments.

However, this study still has several limitations. First, as a cross-sectional study, its findings have limited generalizability, and it cannot establish causality. Future large-sample longitudinal studies are needed to observe the changes in the DTI-ALPS index in PD patients. Second, regarding the measurement methods, the MoCA scale used for cognitive staging may have inaccuracies. Future research should supplement this with more comprehensive neuropsychological assessments or develop specialized scales for evaluating cognitive function in PD patients. Third, DTI technology itself has inherent limitations, such as the crossing-fiber problem. Although we performed rigorous quality control, the manual placement of ROIs may introduce some subjectivity. Future work could combine multiple analytical methods to further explore this direction.

## 5 Conclusion

PD patients exhibited abnormalities in the left DTI-ALPS index and diffusivity, which suggested impaired GS function and white matter damage and were associated with cognitive and motor decline. GS dysfunction and white matter microstructural damage in PD patients collectively contribute to the pathological process of motor and cognitive decline in PD. The left DTI-ALPS index may serve as a biomarker for identifying cognitive decline in PD patients.

## Data Availability

Due to the restrictions on protecting participant privacy and ethical review, the original data of this study can be requested from the corresponding author upon reasonable request (Xiehua Xue, E-mail: f110015@fjtcm.edu.cn).
