# Supplementary material for: The left DTI-ALPS index: a potential glymphatic mediator of cognitive and motor dysfunction in Parkinson’s disease
Source: Front Med (Lausanne). 2025 Nov 28;12:1674718. doi: 10.3389/fmed.2025.1674718 (PMC12698549; doi:10.3389/fmed.2025.1674718)
Supplement: Supplementary file 1 [file Table_1.DOCX]

**The Left DTI-ALPS Index: A Potential Glymphatic Mediator of Cognitive and Motor Dysfunction in Parkinson's Disease**

Yanyan Li^a,c#^, Jian Song^a,b,c#^, Yuqing Zhao^a,b,c^, Zengru Lin^a^, Xingduo Pan^a^, Ming Li^a^,

Xiaoyan Zhou^a^, Zhen Zhang^a^, Wei Wei^a,c^, Xiehua Xue^a,c*^

^a^The Affiliated Rehabilitation Hospital, Fujian University of Traditional Chinese Medicine, Fuzhou, China

^b^Fujian University of Traditional Chinese Medicine, Fuzhou, China

^c^Fujian Key Laboratory of Cognitive Rehabilitation, Fuzhou, China

*Correspondence：

Xiehua Xue

The Affiliated Rehabilitation Hospital, Fujian University of Traditional Chinese Medicine, Fuzhou, China

1. mail: [f110015@fjtcm.edu.cn](mailto:f110015@fjtcm.edu.cn)

^#^These authors contributed equally to this article.

**Supplementary Materials**

**1.MRI scan protocol and analysis**

**1.1 MRI Data Acquisition**

The MRI imaging sequences included T1-weighted imaging (T1WI), T2 fluid-attenuated inversion recovery (T2 FLAIR), and diffusion tensor imaging (DTI). The DTI sequence employed a single-shot echo planar imaging (SS-EPI) technique with 30 acquisition directions and two b-values: b=0 and b=1000 s/mm². Detailed parameters are provided in supplementary materials Table 1.

| **Table 1 The scan parameters of MRI** | | | | | | |
| --- | --- | --- | --- | --- | --- | --- |
| Sequence | TR/ms | TE/ms | Thickness/mm | FOV/mm² | Matrix | Number of layers |
| 3D T1WI | 2200 | 2.48 | 1 | 250×250 | 256×256 | 176 |
| T2 FLAIR | 8000 | 82 | 5 | 220×220 | 233×320 | 22 |
| DTI | 5000 | 69 | 2 | 224×224 | 128×128 | 48 |
| Abbreviations：FOV, Field of View; TR: Repetition Time; TE: Echo Time | | | | | | |

**1.2 Motion Correction and Data Quality Assessment**

We utilized the Eddy tool within the FSL software to simultaneously correct for artifacts from subject head motion and eddy currents induced by gradient switching. This process involved aligning each diffusion-weighted image to a non-diffusion-weighted (b=0) reference image.

To evaluate the effectiveness of the correction and assess data quality, the Eddy quality tool was used to automatically generate a quality control report. This report provided key quantitative metrics, including mean absolute displacement, mean relative displacement, and mean translations and rotations along the x, y, and z axes. These metrics provided an objective basis for assessing data suitability for subsequent analysis.

Furthermore, we established stringent exclusion criteria: data from any subject with a mean relative displacement exceeding 1.0 mm or mean rotation in any direction greater than 0.5 degrees were excluded. This procedure was implemented to minimize the potential confounding effects of head motion on the subsequent group comparisons and correlation analyses.

| **Table 1 Motion Correction and Data Quality Assessment** | | | | |
| --- | --- | --- | --- | --- |
| Item | PD group (n=64) | HC group (n=30) | t/Z | *P* |
| Average abs motion (mm) | 0.54±0.20 | 0.54±0.21 | -0.086 | 0.932 |
| Average rel motion (mm) | 0.23(0.13,0.28) | 0.26(0.20,0.29) | -1.462 | 0.145 |
| Average x translation (mm) | 0.05(-0.11,0.19) | -0.04(-0.21,0.08) | -1.732 | 0.084 |
| Average y translation (mm) | -0.27(-0.35,-0.18) | -0.28(-0.35,-0.24) | -0.690 | 0.494 |
| Average z translation (mm) | 0.13(-0.15,0.31) | 0.23(-0.12,0.42) | -1.480 | 0.140 |
| Average x rotation (deg) | -0.08±0.38 | -0.01±0.31 | -0.973 | 0.333 |
| Average y rotation (deg) | 0.12(-0.13,0.26) | 0.07(-0.10,0.14) | -0.414 | 0.682 |
| Average z rotation (deg) | -0.02±0.34 | -0.02±0.23 | 0.052 | 0.958 |
